# Extracellular Vesicles in Dermatology: Where Are We and What Lies Ahead?

**DOI:** 10.1111/exd.70375

**Published:** 2026-11-02

**Authors:** Ines Martic, Alessandro Paolini, Stefania Paola Bruno, Andrea Gallego, Dorottya Ádám, Sedat Odabas, Şebnem Erçelen Ceylan, Emanuela Camera, Paloma Pérez, Attila Oláh, Andrea Masotti, Mirjana Nacka‐Aleksić, Danuta Gutowska‐Owsiak, Nsrein Ali, Maria Cavinato

**Affiliations:** ^1^ Institute for Biomedical Aging Research University of Innsbruck Innsbruck Austria; ^2^ Center for Molecular Biosciences Innsbruck Austria; ^3^ Research Laboratories Bambino Gesù Children's Hospital‐IRCCS Rome Italy; ^4^ Instituto de Biomedicina de Valencia IBV‐CSIC Valencia Spain; ^5^ Department of Physiology, Faculty of Medicine University of Debrecen Debrecen Hungary; ^6^ Biomaterials and Tissue Engineering Laboratory (bteLAB), Department of Chemistry, Faculty of Science Ankara University Ankara Türkiye; ^7^ Interdisciplinary Research Unit for Advanced Materials (INTRAM) Ankara University Ankara Türkiye; ^8^ Department of Biophysics, Hamidiye Faculty of Medicine University of Health Sciences İstanbul Türkiye; ^9^ Laboratory of Cutaneous Physiopathology San Gallicano Dermatological Institute – IRCCS Rome Italy; ^10^ Institute for the Application of Nuclear Energy (INEP), Department of Reproductive Biology University of Belgrade Zemun Serbia; ^11^ Laboratory of Experimental and Translational Immunology Intercollegiate Faculty of Biotechnology of the University of Gdańsk and the Medical University of Gdańsk, UG Institute of Biotechnology Gdańsk Poland; ^12^ Research Unit of Clinical Medicine, Faculty of Medicine University of Oulu Oulu Finland

**Keywords:** dermatology, extracellular vesicles, inflammation, intercellular communication, skin ageing, skin biology, tumour microenvironment, wound healing

## Abstract

Extracellular vesicles (EVs) have emerged as key players in skin biology, mediating intercellular communication across diverse physiological and pathological contexts. In this review, we critically assess the current understanding of EVs in dermatology, highlighting their roles in skin homeostasis, ageing, inflammation, wound healing and cancer. We explore the mechanistic contributions of EVs derived from skin, emphasizing their influence on immune modulation, senescence and tissue regeneration. We also examine their potential as diagnostic biomarkers and therapeutic agents, while addressing key technical and conceptual challenges that limit clinical translation. Drawing on collaborative insights from the COST Action NETSKINMODELS, we provide a set of recommendations to advance the field, including standardizing separation protocols, improving functional characterization and integrating with emerging technologies such as microneedles and 3D bioprinting. Together, these perspectives outline a strategic framework for unlocking the diagnostic and therapeutic potential of EVs in dermatological research and clinical care.

**Abbreviations**
AD: atopic dermatitis
ADSC: adipose‐derived stem cell
AKT: protein kinase B
CPD: cyclobutane pyrimidine dimer
cSCC: cutaneous squamous cell carcinoma
ECM: extracellular matrix
EGFR: epidermal growth factor receptor
EP: extracellular particle
ERK: extracellular signal‐regulated kinase
EV: extracellular vesicle
FLG: filaggrin
GelMA: methacrylated gelatin
HUVEC: human umbilical vein endothelial cell
ICAM‐1: intercellular adhesion molecule 1
IL: interleukin
JNK: c‐Jun N‐terminal kinase
lncRNA: long non‐coding RNA
MHC: major histocompatibility complex
miRNA: microRNA
MN: microneedle
MSC: mesenchymal stem cell
mtDNA: mitochondrial DNA
NVEP: non‐vesicular extracellular particle
O‐PTIR: optical photothermal infrared (imaging)
PEGDA: poly(ethylene glycol) diacrylate
PLA2: phospholipase A2
ROS: reactive oxygen species
SASP: senescence‐associated secretory phenotype
SA‐β‐Gal: senescence‐associated β‐galactosidase
sEV: small extracellular vesicle
STING: stimulator of interferon genes
TGF‐β: transforming growth factor beta
Th: T helper (cell)
TLR: Toll‐like receptor
TSLP: thymic stromal lymphopoietin
UV: ultraviolet
UVA: ultraviolet A
UVB: ultraviolet B

## 1 Introduction

The appearance and function of the skin depend on complex interconnections and communication among all skin cells and compartments. Extracellular particles (EPs), which include both vesicular and non‐vesicular EPs (NVEPs), have been described as important players in intercellular communication [1, 2, 3, 4]. Among them, extracellular vesicles (EVs) are increasingly recognized for their involvement in skin homeostasis and pathology [5, 6]. EVs are membrane‐bound, nano‐sized vesicles released into the extracellular space. According to the most recent guidelines of the ‘Minimal Information for Studies of Extracellular Vesicles’ publication [7], EVs can be roughly classified into different subtypes based on their size and biogenesis [7]. In contrast, NVEPs are non‐membrane‐bound particles, including protein aggregates such as exomeres, nucleic acid complexes and lipoproteins, that also contribute to intercellular communication [7]. EV nomenclature is ideally based on biogenesis, but in practice the distinction between different types of EVs is not always precise, particularly when separation relies solely on size or density rather than direct markers of origin, and we have adopted this terminology pragmatically to reflect how individual studies describe their preparations. In line with MISEV2023, in this review we use the umbrella term ‘EVs’ when vesicles are of unknown or mixed origin, and ‘small EVs (sEVs)’ for vesicles typically < 200 nm, often enriched by high‐speed centrifugation or density‐based methods [7]. Where studies specifically address plasma‐membrane‐derived larger EVs (historically termed microvesicles, microparticles or ectosomes), we refer to them as ‘large EVs (lEVs)’, acknowledging their distinct biogenesis and pharmacological tractability.

EVs are secreted by all cell types and can be found throughout the body in tissues and biological fluids, allowing for widespread distribution and function across different tissues and organs [8, 9]. EVs carry a variety of bioactive molecules, including proteins, lipids and nucleic acids, and their molecular composition contributes to their functional diversity. The principal EV biogenesis pathways, molecular components and modes of interaction with recipient cells are summarized in Figure 1. EVs influence the extracellular environment of tissues through molecular interactions that can modulate cellular behaviour, extracellular matrix (ECM) composition and immune responses. EVs exert their effects on recipient cells through interactions of EV surface molecules with cellular receptors and, in some contexts, by transfer of luminal cargo after internalization, thereby influencing gene expression, proteostasis, and signalling pathways [10]; however, the relative contribution and frequency of bona fide luminal cargo delivery versus surface or endosomal signalling remain a matter of active investigation. Hence, EVs are pivotal in coordinating cellular physiological functions and responses to environmental challenges. Understanding the roles and mechanisms involving EVs in skin homeostasis, ageing and disease can provide valuable insights into novel therapeutic approaches for various skin conditions.

**FIGURE 1:**
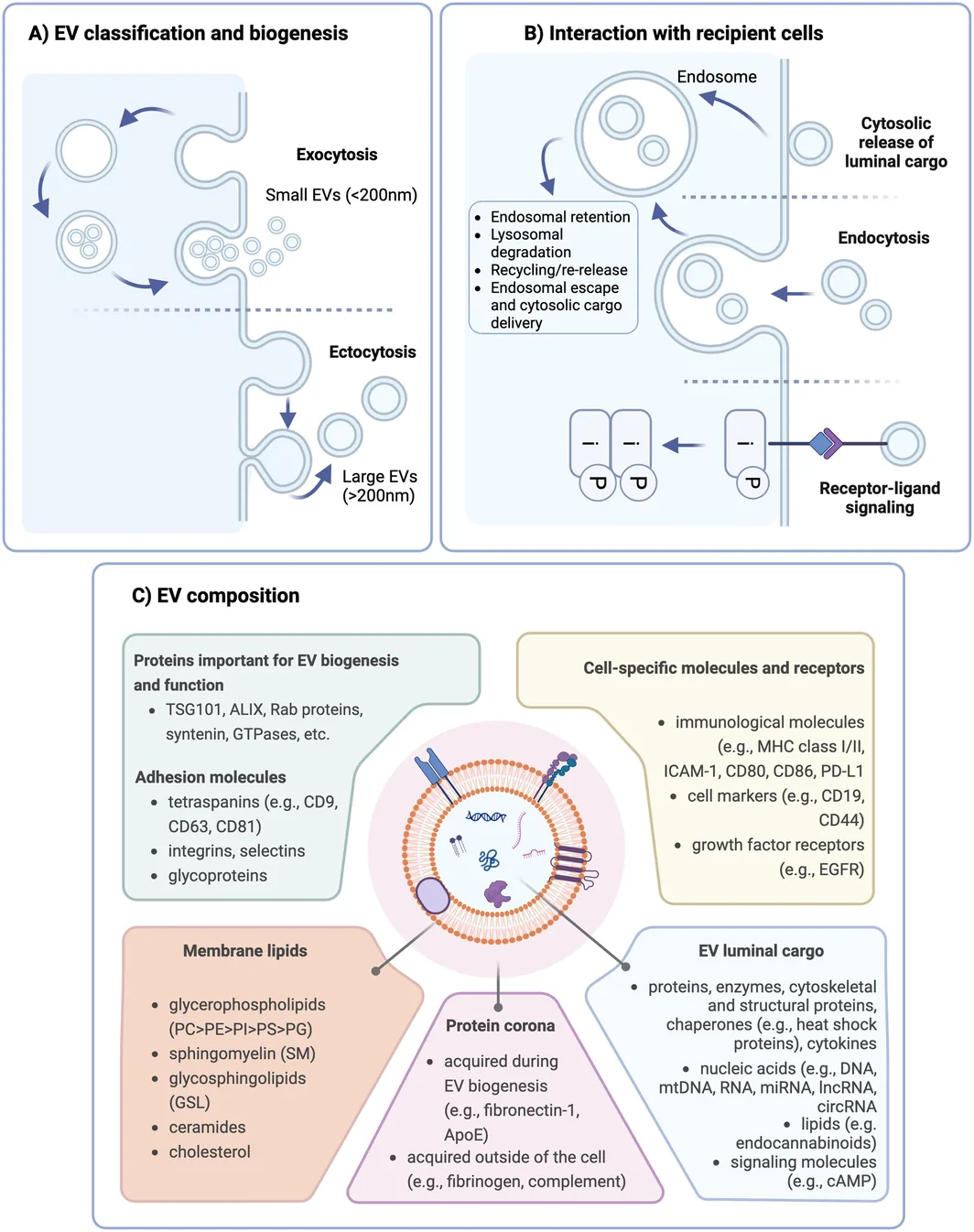
Classification, molecular composition and interactions of extracellular vesicles with recipient cells. (A) EVs arise through several biogenetic routes. Endosomal maturation and fusion of multivesicular bodies with the plasma membrane release populations enriched in small EVs, whereas outward budding of the plasma membrane generates populations enriched in medium/large EVs. Because EV biogenesis is rarely demonstrated directly, size‐based operational terminology does not establish the subcellular origin of an EV preparation. (B) EVs may influence recipient cells through receptor–ligand interactions at the cell surface or following internalization. Internalized EVs may remain within endosomal compartments, undergo lysosomal degradation, or be recycled or re‐released. In some experimental contexts, direct membrane fusion or endosomal escape may permit cytosolic delivery of luminal cargo; however, cellular association or internalization alone does not demonstrate functional cargo delivery. (C) EVs contain membrane lipids and proteins, cell‐specific surface molecules, surface‐associated proteins and protein‐corona components, and luminal cargo comprising proteins, nucleic acids, lipids, metabolites and signalling molecules. Their composition varies according to cellular source, physiological state and separation method. Created with BioRender.com. Cavinato, M. (https://BioRender.com/redbfvh) is licensed under CC BY 4.0.

This review synthesizes current knowledge in the field, emphasizing the potential of EVs as biomarkers and diagnostic tools, their roles in immunomodulation, inflammation and allergic disorders, their involvement in skin cancer and the tumour microenvironment, and their contribution to wound healing and skin regeneration. These discussions will highlight the critical role of EVs in both maintaining skin health and contributing to the development of pathological conditions. We additionally translate MISEV2023 and related methodological guidance into practical minimum‐reporting recommendations for skin‐derived EV research (Box 1) [7, 11] and distinguish among in vitro, animal, observational human and interventional clinical evidence.

Several reviews have summarized the roles and potential application of EVs in skin physiology, ageing, disease and regenerative processes [5, 6, 12]. The distinctive contribution of the present review is a cross‐cutting framework that connects the skin exposome, cellular senescence and EV biology; considers EVs as context‐dependent mediators that may either propagate tissue damage or support repair; and evaluates methodological rigour and translational readiness alongside biological findings. We additionally translate MISEV2023 into practical minimum‐reporting recommendations for skin‐derived EV research and distinguish among in vitro, animal, observational human, and interventional clinical evidence.

BOX 1 **Minimal recommendations for research on skin‐derived EVs.**

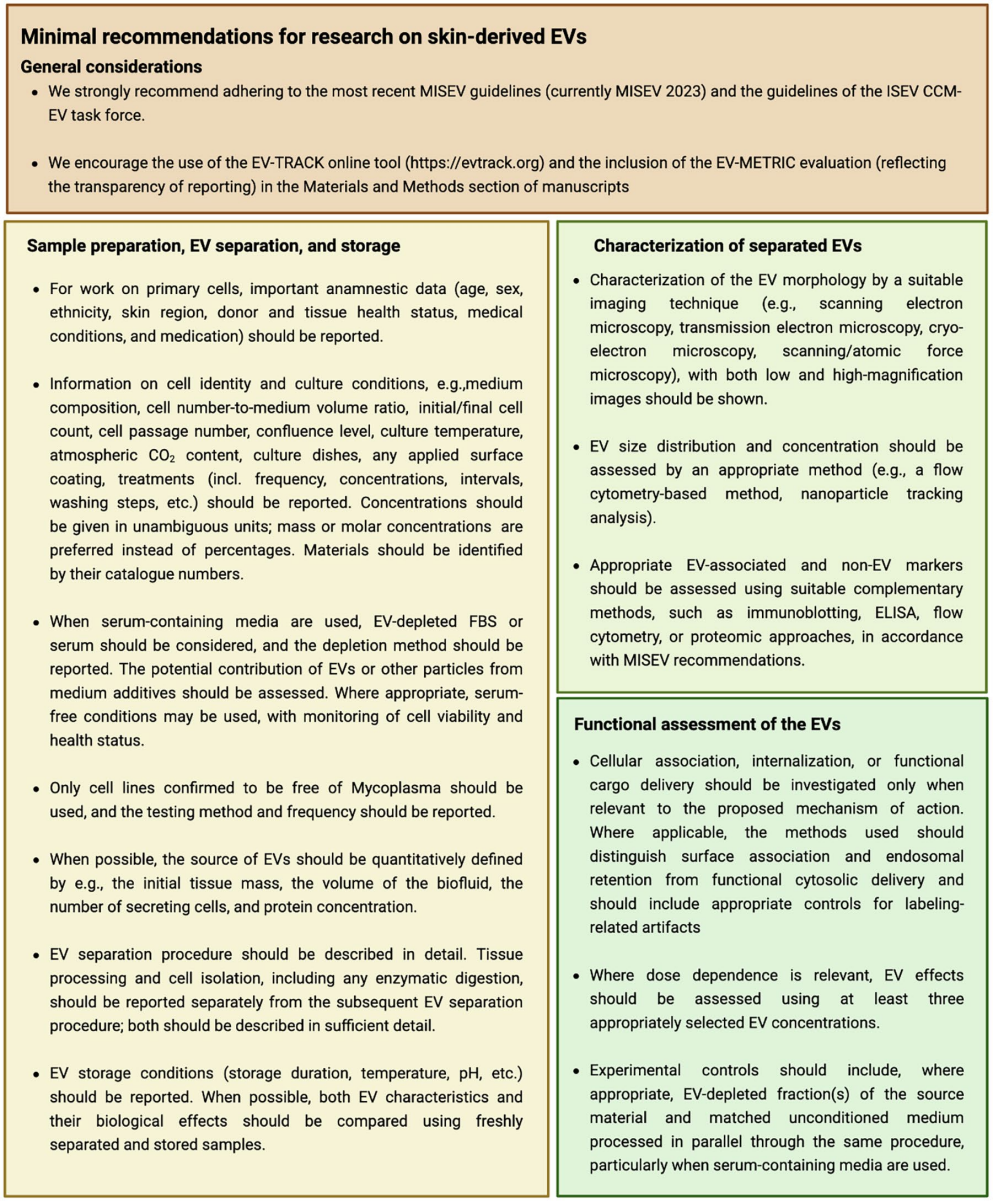

## 2 The Role of EVs in Skin Homeostasis

EVs play a crucial role in maintaining skin homeostasis by regulating structural integrity, intercellular communication and signalling. Keratinocyte‐derived EVs are particularly significant because they modulate processes such as pigmentation by releasing miRNA‐containing EVs that regulate melanin production, thereby affecting skin colour and UV protection [12, 13]. Additionally, keratinocyte‐derived EVs mediate communication with fibroblasts, influencing gene expression, differentiation, and signalling pathways to support skin homeostasis and repair mechanisms. For example, keratinocyte‐derived EVs activate ERK1/2 and JNK pathways, promoting fibroblast migration and ECM remodelling, which are critical for effective wound healing and tissue regeneration [14].

Additionally, keratinocyte‐derived EVs play a vital role in modulating cutaneous immunity, influencing skin responses to environmental stressors and pathogens. These vesicles facilitate communication between keratinocytes and various immune cells, thereby participating in both innate and adaptive immune responses. For instance, UVB‐irradiated keratinocyte‐derived EVs were shown to induce a STING (stimulator of interferon genes)–mediated inflammatory response in dermal macrophages, thereby heightening skin inflammation. This response is characterized by the recruitment of inflammatory cells and the production of pro‐inflammatory cytokines, that is, IL‐6 and IL‐8, which are crucial for initiating immune responses [15, 16]. On the other hand, by stimulating the production of anti‐inflammatory cytokines or promoting the apoptosis of activated immune cells, keratinocyte‐derived EVs may also have immunomodulatory effects, preventing chronic inflammation and possibly permanent skin damage [15, 17].

Furthermore, EVs contribute to the skin barrier function by delivering essential lipids and proteins necessary for the integrity of the stratum corneum [6]. It has recently been shown that sEVs secreted by keratinocytes remove excess free, unsequestered filaggrin accumulating in cells before the formation of storage keratohyalin granules [18]. Unsequestered filaggrin in the cytosol is cytotoxic to cells [19], and inhibition of sEV secretion by keratinocytes leads to premature cell death [18]. Hence, EVs appear to be involved in a fundamental cellular mechanism that enables the progression of keratinocyte differentiation (and consequent stratification), thereby supporting skin barrier formation. Importantly, even microbiota‐derived EVs also influence the behaviour of skin cells, thereby modulating skin health and disease [20, 21, 22].

## 3 The Role of EVs in Skin Ageing

As skin ages, it loses elasticity, develops uneven pigmentation, wrinkles and becomes more vulnerable to chronic low‐grade inflammation and skin cancer. Traditionally, skin ageing is thought to result from both intrinsic (chronological, genetically programmed) and extrinsic (environmentally driven) factors that accumulate over time [23]. However, contemporary concepts introduce a more holistic ‘skin exposome’ framework [24], which captures the cumulative impact of skin lifetime exposures, including solar radiation (UV, visible light and infrared radiation) [25, 26, 27], atmospheric pollution (particulate matter‐PM, ozone) [26, 28], cigarette smoke and lifestyle factors [29, 30] and emerging pollutants as micro‐ and nanoplastics [31, 32], which synergistically accelerate the molecular pathways of ageing.

A major hallmark of skin ageing is the accumulation of senescent cells in the skin compartments. These cells are characterized by stable cell cycle arrest, retained metabolic activity and secretion of a broad spectrum of pro‐inflammatory cytokines, chemokines and proteases collectively known as the senescence‐associated secretory phenotype (SASP) [33]. Apart from their role in skin ageing as a critical communication tool among cells, skin‐derived EVs have been recognized as components of the SASP and may influence age‐related changes [34].

Among extrinsic factors, UV radiation remains the predominant contributor to skin ageing, accounting for most of the oxidative stress and DNA damage in sun‐exposed skin [35], while oxidative stress has been considered central to collagen degradation and ECM remodelling (reviewed in [36]). Moreover, chronic UVA/UVB exposure damages nuclear and mitochondrial DNA both directly, through the formation of cyclobutane pyrimidine dimers (CPDs) and other photoproducts, and indirectly, through reactive oxygen species (ROS) generation, as shown in HaCaT keratinocytes and ex vivo skin explants [37]. In addition, UVA radiation has been shown to activate the MAPK pathway and upregulate the expression of Cathepsin K in human dermal fibroblasts, which degrades elastin and induces solar elastosis [38].

In response to such UV‐induced stress, skin cells alter their EV secretion and cargo composition and act as vehicles for propagating senescence‐associated changes, ECM remodelling, and chronic low‐grade inflammation across the ageing skin microenvironment [39]. In line with this notion, it was shown that sEVs carrying CPD photoproducts, released in a caspase‐dependent manner from UVB‐irradiated HaCaT keratinocytes and skin explants, can be engulfed by neighbouring and distant keratinocytes, effectively spreading the molecular signature of UV damage [40]. Similarly, EVs from fibroblasts exposed to UVB radiation were shown to be enriched in miR‐22‐5p and promote a photoageing phenotype in previously unexposed recipient cells. Conversely, silencing this miRNA in EVs ameliorated photoageing in vitro and in vivo, demonstrating that ageing signals can be ‘packaged’ into EVs and disseminated between cells [41]. Senescent fibroblasts were also found to secrete high levels of EVs due to increased neutral sphingomyelinase (nSMase) activity, oxidative stress and altered lysosomal function [42]. nSMase catalyses the conversion of sphingomyelin to ceramide and phosphocholine, which are key building blocks of the epidermal barrier [43], and its upregulation may also promote skin inflammation [44]. In line with these findings, treatment of UVB‐irradiated human keratinocytes with the acid sphingomyelinase (ASM) inhibitor imipramine altered the release of extracellular DNA containing UV photoproducts in sEVs, indicating that ASM activity contributes to the packaging and export of UV‐damaged DNA in small EVs [45]. Together with data from nSMase‐dependent EV release in senescent fibroblasts, these observations suggest that distinct sphingomyelinases regulate both the biogenesis and damage‐associated cargo of skin‐derived EVs in response to UV stress.

Also, EVs derived from senescent fibroblasts were shown to highly express the pro‐inflammatory cytokine IL‐6, thus contributing to the inflammaging processes and impairing keratinocyte differentiation and barrier function [42].

Additionally, epidemiological and mechanistic studies show that PM and ozone exposure may aggravate a range of skin disorders, including accelerated ageing, atopic dermatitis and aberrant pigmentation, by activating molecular pathways that closely parallel those triggered by UV radiation, that is, oxidative stress, barrier dysfunction and inflammation [46, 47]. Additionally, evidence from other systems indicates that environmental pollutants, including PM, tobacco smoke, and chemical contaminants, modulate EV release rate and microRNA/protein luminal cargo composition, which may contribute to the development of various pathologies by triggering oxidative stress, coagulation and inflammation [48, 49].

Recently, it has been proposed that EV‐mediated mitochondrial disposal released by senescent fibroblasts may serve as an alternative quality control mechanism to maintain cellular homeostasis in response to impaired autophagy and mitophagy [50]. Senescent fibroblasts also release EVs containing miR‐15b‐5p and miR‐30a‐3p, which have an anti‐apoptotic effect, possibly contributing to the accumulation of senescent cells in the skin [51]. Furthermore, fibroblast‐derived sEVs were reported to modulate keratinocyte migration and differentiation in 2D and 3D models in a manner that varied with both fibroblast sEV donor age and recipient keratinocyte donor age. In this study, cel‐miR‐39, a non‐mammalian miRNA, was used as a spike‐in tracer to track the transfer of EV‐associated RNA to recipient keratinocytes; however, its detection in recipient‐cell preparations does not by itself establish cytosolic delivery, and the study did not attribute the observed functional effects specifically to this foreign miRNA [9].

Similarly, UVA‐induced plasma membrane damage in melanocytes triggers the release of EVs enriched with lysosomal proteins, which are rapidly internalized by neighbouring keratinocytes [52]. These EVs enhance keratinocyte proliferation and migration while concurrently increasing resistance to apoptosis by upregulating miR‐21 expression in recipient cells [53]. These studies highlight EVs as important regulators of skin ageing by transmitting senescence‐ and stress‐associated molecular signals that reshape recipient cell behaviour and contribute to alterations in skin homeostasis.

Given the significant role of EVs in modulating cellular processes during cellular senescence and skin ageing, we believe that targeting EV pathways could offer a novel therapeutic strategy against age‐related pathologies. In that vein, mesenchymal stem cell‐ and other stem cell–derived EVs are increasingly investigated as anti‐ageing and regenerative agents for skin, underscoring that the same vesicular communication system implicated in propagating damage can, depending on its source and cargo, also be harnessed to counteract it [54]. For example, EVs derived from adipose‐derived stem cells (ADSCs) enriched in miR‐1246 were reported to attenuate UVB‐induced photoageing by suppressing of GSK3β and enhancing autophagic flux, thereby reducing oxidative stress, MMP‐1 expression, and DNA damage, while promoting cell viability and Type I procollagen synthesis [55]. Furthermore, in one clinical study, intradermal administration of autologous adipose tissue‐derived nanovesicles significantly improved multiple signs of skin ageing, including wrinkles, hydration, softness and overall skin quality, with high patient satisfaction and no apparent adverse effects, highlighting their potential as a minimally invasive skin rejuvenation therapy [56].

In conclusion, the presented findings position EVs as key molecular mediators at the interface between environmental exposure, cellular senescence, and skin ageing. However, a deeper understanding of how environmental stressors reshape EV biogenesis and cargo, and how these vesicles subsequently influence cellular communication within the ageing skin microenvironment is essential for developing these targeted interventions.

## 4 The Role of EVs in Skin Cancer and Tumour Microenvironment

The rising incidence and mortality rates of skin cancers highlight the need for a deeper understanding of how these cancers develop and how tumour cells influence surrounding tissues to promote their growth [57]. EV‐mediated communication has been shown to be involved in shaping the tumour microenvironment and thereby influencing tumour progression and metastasis formation in multiple systems [58, 59, 60, 61]. In light of this, it is easy to understand that EVs have emerged as a promising target in clinical dermatology as well.

Melanoma, a skin cancer originating from melanocytes, has been extensively studied for its release of EVs, which play a crucial role in tumour progression [62]. Research indicates that melanoma‐derived sEVs separated by multiple steps of differential centrifugation tended to be larger (however, the difference was not statistically significant as compared to primary melanocytes) and contain more mRNAs encoding proteins associated with melanoma progression, such as DNA topoisomerase I [63, 64]. Melanoma‐derived EVs may promote tumour angiogenesis and/or metastasis. Specifically, it has been reported that melanoma cell‐derived sEVs carry inflammatory cytokines and angiogenic factors that facilitate neoangiogenesis, distinguishing them from EVs derived from normal primary melanocytes [63]. Additionally, several lines of evidence argue that, upon entering circulation, EVs from primary melanoma and from cancer‐associated fibroblasts are transferred to metastatic sites, where they may promote vascular permeability and attract immune cells, crucial steps in the formation of pre‐metastatic niches [65, 66, 67]. Moreover, melanoma‐derived sEVs significantly alter the differentiation and composition of immune‐cell populations, particularly T cells, suggesting that these EVs can influence both the local tumour microenvironment and the systemic immune response [68]. A key challenge in melanoma research is distinguishing between melanosomes and EVs, as they share similar properties and markers, complicating the accurate characterization of EVs in melanoma development and progression. However, one could argue that melanosomes themselves constitute a specialized subpopulation of EVs, uniquely defined by their cargo—melanin [69, 70]. This perspective shifts the focus from differentiation to integration, recognizing melanosomes as an EV subtype with distinct functional and biological significance in melanoma biology.

Although research on the role of EV release in melanoma progression remains limited, growing evidence highlights the importance of cells within the tumour microenvironment. For example, adipocytes adjacent to melanoma cells have been shown to release EVs carrying β‐catenin, which downregulates p16 expression in melanoma cells, thereby promoting cancer progression. This finding aligns with epidemiological data indicating that obese individuals are at a higher risk of developing skin cancer [71].

The role of EVs in skin cancers such as cutaneous squamous cell carcinoma (cSCC) remains largely underexplored. In cSCC, desmoglein‐2 overexpression has been shown to significantly enhance EV release, a finding that correlates with tumour burden in xenograft models [72]. Additionally, circulating EVs from cSCC patients contain distinct RNA species, including circ‐CYP24A1, whose inhibition has been found to reduce the proliferation, migration, and invasion of A431 epidermoid carcinoma cells [73]. Furthermore, cSCC‐derived EVs are enriched in proteins involved in TGF‐β signalling, which can induce fibroblasts in the tumour microenvironment to transform into cancer‐associated fibroblasts. These cells, in turn, contribute to tumour progression by remodelling the ECM and promoting epithelial‐mesenchymal transition [74].

In conclusion, EVs are emerging as key players in skin cancer progression and present promising avenues for early tumour detection, novel therapeutic targets, and a deeper understanding of tumour‐immune interactions. Research focusing on the distinct contributions of EVs in melanoma and other skin cancers could lead to significant breakthroughs in the fight against these cancers. However, the field could clearly benefit from more rigorous standardization and transparent reporting of the separation of EVs. Across melanoma and cSCC, a recurring theme is that tumour‐derived EVs remodel the microenvironment by reprogramming stromal fibroblasts, adipocytes, endothelial cells, and immune populations. However, many melanoma and cSCC EV studies rely on a limited number of cell lines, heterogeneous separation protocols, and variable marker characterization, which complicates cross‐study comparisons and may contribute to discrepancies in reported EV size distributions, cargo profiles, and functional effects. Larger patient cohorts with standardized EV phenotyping and parallel analysis of non‐EV fractions will be essential to resolve conflicting findings and to establish which EV‐associated signatures truly predict disease stage or treatment response.

## 5 The Role of EVs in Skin Immunomodulation and Inflammation

EVs play a pivotal role in skin immunomodulation and inflammation by mediating bidirectional communication between immune and non‐immune cells. This perpetuates a feedforward inflammatory loop that drives the onset and progression of inflammatory and immune‐mediated skin diseases [72, 75, 76, 77].

Atopic dermatitis (AD), a prevalent chronic relapsing inflammatory disease, is characterized by skin barrier dysfunction, in which filaggrin (*FLG*) loss‐of‐function mutations, dysbiosis—often due to enhanced colonization with *Staphylococcus aureus* (*S. aureus)—and* an exaggerated Type 2 immune/inflammatory response play key roles [78]. Keratinocytes with reduced or absent filaggrin expression secrete sEVs that are less likely to be taken up by cells, potentially affecting their cargo transfer [79]. sEVs derived from filaggrin‐insufficient keratinocytes also promote a Type 2 proallergic response and deliver lipid antigens to CD1a‐restricted T cells, triggering Th2‐biassed inflammation [80]. Also, keratinocytes use sEVs to export excess FLG and prevent cell death and epidermal barrier dysfunction, a mechanism that can be hijacked by *S. aureus* via TLR‐2 with potential to promote bacterial growth and consequently exacerbate inflammation [18].

*S. aureus* ‐derived EVs (^S.aureus^EVs) are causally related to AD pathogenesis, triggering the release of inflammatory cytokines such as IL‐6, thymic stromal lymphopoietin, and macrophage inflammatory protein by dermal fibroblasts [20, 81]. In mice, topical application of ^S.aureus^EVs induced AD features such as epidermal hyperplasia, immune cell infiltration, and elevated Th1 and Th17 cytokine levels [20]. Further, keratinocytes exposed to ^S.aureus^EVs secrete IL‐6 and IL‐8, amplifying inflammation and promoting neutrophil and macrophage recruitment [20, 75]. ^S.aureus^EVs also contribute to antibiotic resistance, biofilm formation, and overall compromised epidermal barrier function [75, 77]. Also, EVs derived from the AD‐associated skin fungus *Malassezia sympodialis* can damage the epidermal barrier and increase peripheral IL‐4 and TNF‐α production, contributing to Type 2 responses [82].

In psoriasis, characterized by epidermal hyperproliferation and T helper (Th)1/Th17 immune responses [83], keratinocyte‐derived EVs were shown to contribute to the inflammatory environment by promoting a positive feedback loop between keratinocytes and immune cells. When taken up by Th cells, EVs from psoriatic keratinocytes promote their differentiation toward a Th1 and/or Th17 phenotype and the production of pro‐psoriatic cytokines [84]. In response to the psoriatic trigger IL‐17A, keratinocytes release EVs, which are internalized by neutrophils, triggering the release of neutrophil extracellular traps, IL‐6, IL‐8, and TNF‐α [85]. EVs secreted by macrophages further promote keratinocyte proliferation and disease progression [72].

Interestingly, phospholipase A2 (PLA2), an enzyme highly expressed in psoriatic lesions, is found in sEVs produced by mast cells and linked to lipid‐specific T‐cell inflammatory skin responses [86]. Consistently, circulating and skin‐derived T cells from psoriatic patients showed heightened sensitivity to PLA2‐generated neoantigens compared with those from healthy controls. Since CD1a is also constitutively overexpressed in psoriatic lesions, targeting EV PLA2 and/or CD1a could represent a potential therapeutic strategy.

Taken together, EVs from keratinocytes, immune cells, and microbes appear to drive a feed‐forward inflammatory loop in AD and psoriasis, and several studies converge on EV‐mediated IL‐6/IL‐8 and Th1/Th17 polarization as shared mechanisms. At the same time, differences in patient cohorts, EV separation and labelling methods, and the frequent use of lipophilic dyes or ultracentrifugation pellets without non‐EV controls introduce potential biases that can inflate perceived effect sizes. Future work using rigorously characterized EV preparations, standardized clinical endpoints, and appropriate negative controls will be crucial to reconcile these findings and to translate EV‐based biomarkers or interventions into practice.

Apart from playing key roles in the pathogenesis of both AD and psoriasis, EVs have the potential to serve as biomarkers and therapeutic tools in inflammatory skin diseases by modulating immune responses and pathogen survival, promoting healing, and delivering drugs. Future research should focus on enhancing the therapeutic potential of EVs by improving targeting mechanisms, optimizing their bioengineering, and exploring these particles as carriers of therapeutic cargoes.

## 6 The Role of EVs in Wound Healing and Skin Regeneration

Wound healing and skin regeneration are vital biological processes that restore the integrity and function of the skin following injury. These complex mechanisms involve a series of overlapping phases, each playing a crucial role in tissue repair [87]. Recent studies have revealed that skin‐derived EVs are pivotal in these mechanisms, owing to their ability to modulate a wide range of biological functions, including inflammation, cell migration, and tissue remodelling [15, 88]. The initial phase of wound healing is characterized by inflammation, which is essential for pathogen defence and tissue repair but can be detrimental if prolonged or dysregulated [89]. Skin‐derived EVs have been shown to regulate this inflammatory response. For example, activated keratinocytes secrete EVs that contain proteins enriched in immune‐related pathways, which can modulate immune cell activity at the wound site. These EVs can either promote or suppress the activation of immune cells, such as macrophages [88] and neutrophils [90], which are key players in both the inflammatory and tissue remodelling phases of wound healing. By influencing immune cell polarization, skin EVs help resolve acute inflammation and prevent chronic inflammation, which can delay healing.

For wound healing to progress, cells must migrate to the injury site, proliferate, and differentiate to restore tissue integrity. EVs from skin cells, such as fibroblasts, keratinocytes, and endothelial cells, can facilitate these processes. For instance, keratinocyte‐derived EVs promote migration and proliferation of keratinocytes, which are essential for re‐epithelialization of the wound surface [91]. Similarly, keratinocyte‐ and fibroblast‐derived EVs contain growth factors, such as TGF‐β, that stimulate fibroblast migration and proliferation, which are key for collagen production and ECM formation during the wound‐healing process [92, 93]. Moreover, EVs play a role in recruiting endothelial cells to the wound site, which is crucial for angiogenesis and for providing an adequate supply of oxygen and nutrients to the wound, both of which are necessary for tissue regeneration. EVs containing pro‐angiogenic factors, such as vascular endothelial growth factor, have been shown to accelerate angiogenesis, thereby enhancing tissue regeneration and healing [93].

In wound healing, proper ECM remodelling is vital for restoring tissue function. Skin cell‐derived EVs may also contain matrix metalloproteinases that degrade the ECM, facilitating remodelling of the wound site [94]. These enzymes also aid in clearing damaged ECM components, creating space for the deposition of new ECM materials and promoting tissue repair. Furthermore, EVs from fibroblasts and other skin cells carry signalling molecules that influence the synthesis and maturation of collagen, a key component of the ECM, ultimately restoring the structural integrity of the skin [93, 95].

To summarize, skin‐derived EVs are critical regulators of wound healing and skin regeneration. By regulating inflammation, cell migration, proliferation, and ECM remodelling, they orchestrate the complex processes that restore tissue integrity after an injury. Their potential as a therapeutic tool for enhancing wound healing in both acute and chronic wounds offers exciting opportunities to advance regenerative medicine. Nonetheless, most data come from murine models and in vitro systems, with relatively small and heterogeneous clinical cohorts, and differences in EV sources and formulations hinder direct comparison between studies. Addressing these design biases through harmonized protocols and adequately powered trials will be important for determining which EV‐based strategies are genuinely effective for chronic wound management.

## 7 Context‐Dependent Roles of EVs in Skin Biology and Disease

Across skin homeostasis, ageing, inflammation, cancer, and wound repair, EV‐associated effects converge on a limited set of shared biological processes: modulation of immune‐cell activation, remodelling of the extracellular matrix and barrier, regulation of cellular stress responses, and alteration of cell migration, proliferation, and differentiation [6, 62, 75]. The direction of these effects is context‐dependent rather than intrinsically beneficial or harmful. EV preparations from healthy or regenerative cell sources are frequently associated with tissue repair, whereas EVs produced under inflammatory, senescent, or malignant conditions may amplify tissue dysfunction. This source‐ and state‐dependence provides a unifying framework for comparing otherwise disease‐specific observations.

Apparently conflicting findings cannot always be interpreted as genuine biological disagreement because studies differ considerably in donor characteristics, cell models, EV size fractions, separation methods, dose normalization, exposure time, and functional endpoints [7]. Detection of a candidate cargo molecule in an EV preparation, together with cellular association or internalization of that preparation, does not by itself demonstrate cytosolic delivery of luminal cargo. Mechanistic interpretation therefore requires adequate exclusion of co‐separated non‐vesicular material and direct evidence of membrane fusion, endosomal escape, or functional cargo delivery. Rigorous reporter‐based studies have shown that cytosolic delivery by unmodified EVs may be inefficient or below the detection threshold in the experimental systems examined [96, 97]. Associations between a candidate EV cargo molecule and a recipient‐cell phenotype should consequently be distinguished from evidence demonstrating that the molecule is necessary, sufficient, and functionally delivered.

The available evidence is further limited by its strong preclinical skew. Much of the literature relies on immortalized cell lines, single‐source EV preparations, small animal studies, and incompletely reported donor variables. Independent replication, blinded outcome assessment, preregistration, and adequately powered human cohorts remain uncommon. Positive‐result and publication bias may consequently inflate the apparent consistency and translational promise of EV‐based approaches. These limitations do not negate the biological relevance of EVs, but they determine the confidence that can be assigned to individual claims and define the evidence required for clinical translation.

These context‐dependent roles of EVs across skin ageing and senescence, inflammatory skin diseases, skin cancer, and wound repair, together with their principal applications and requirements, are summarized in Figure 2.

**FIGURE 2:**
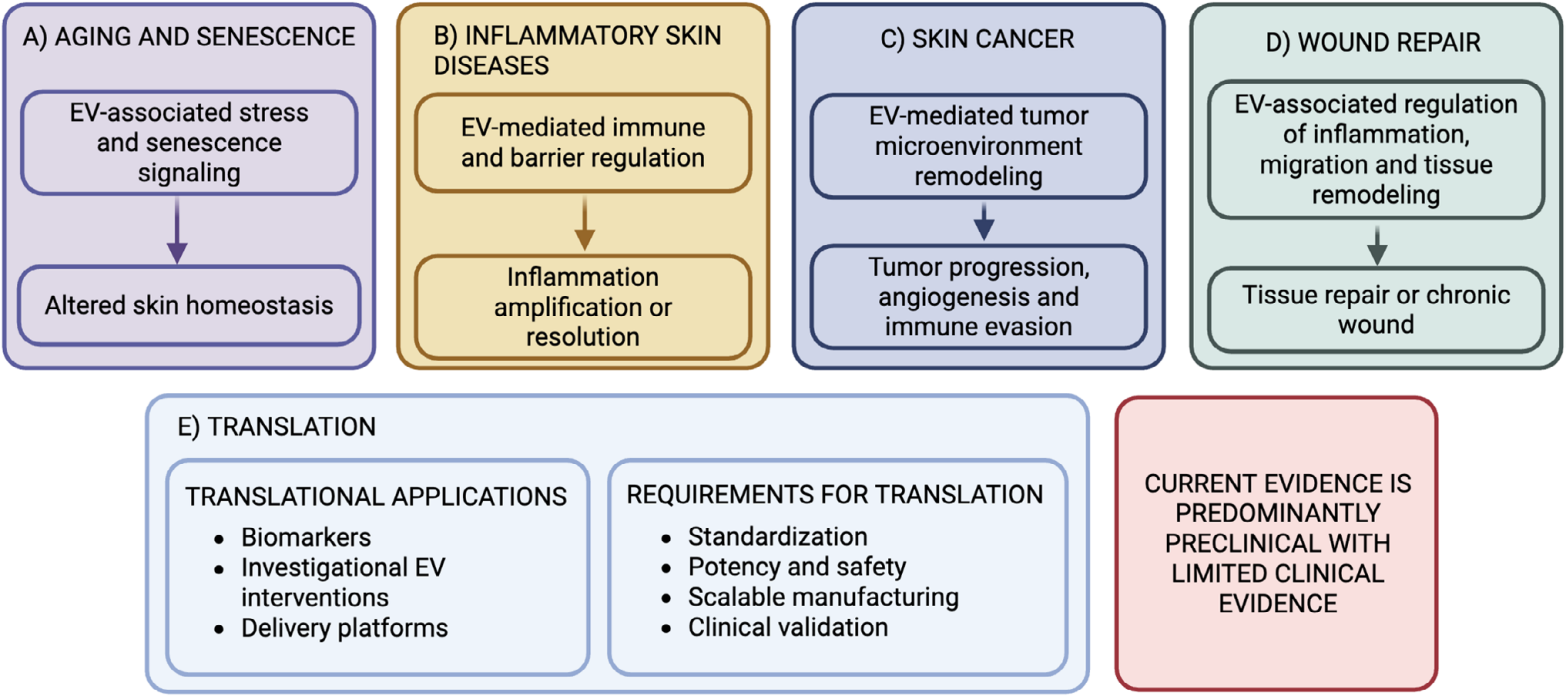
Context‐dependent roles and translational applications of extracellular vesicles in dermatology. Across skin ageing and senescence, inflammatory skin diseases, skin cancer, and wound repair, extracellular vesicle (EV)‐associated effects converge on regulating cellular stress responses, immune and barrier functions, intercellular communication and tissue remodelling. Depending on their cellular source and physiological or pathological context, these effects may contribute to altered skin homeostasis, inflammation amplification or resolution, tumour progression, tissue repair or chronic pathology. Proposed translational applications include EV‐associated biomarkers, investigational EV‐based interventions and delivery platforms. Their clinical development requires standardized characterization, validated potency and safety assessment, scalable manufacturing and clinical validation. Current evidence remains predominantly preclinical, with limited early clinical evidence. Created in BioRender. Cavinato, M. (2026) https://BioRender.com/e34u6sy.

## 8 EVs As Biomarkers/Diagnostic Tools in Skin Diseases

EVs are promising biomarkers and diagnostic tools in dermatology due to their ability to carry molecular signatures reflective of their cells of origin. Their potential for non‐invasive and early diagnosis spans both healthy skin (e.g., assessing barrier integrity) and disease states, including inflammatory, autoimmune, and cancerous conditions. Among accessible biofluids, sweat is of dermatological interest because it can be collected non‐invasively at the skin surface. Proof‐of‐concept studies using clinical‐grade patches identified metabolic differences in sweat EV‐enriched preparations following heat exposure and during exercise and recovery [98, 99]. These findings remain preliminary, and their clinical translation will require standardized sweat collection, EV separation and characterization, and validation in adequately powered dermatological cohorts.

Additionally, EVs show promise in the early detection of skin cancers such as melanoma, basal cell carcinoma, and cSCC. Tumour‐derived EVs often exhibit aberrant protein and miRNA profiles, reflecting malignancy. For example, melanoma‐derived EVs contain specific molecules, such as S100B and Melanoma Inhibitory Activity, which are upregulated in tumour cells and detectable in the blood of patients, aiding early diagnosis and monitoring [100, 101]. Furthermore, the amount of programmed death‐ligand‐1 on EVs positively correlates with the clinical response to immune checkpoint inhibitors, suggesting that EVs could help identify patients most likely to benefit from such treatments [101].

A study of plasma EVs in melanoma patients revealed elevated fatty acid levels in Stage II patients, while stages III–IV showed reduced levels of the Ras‐related protein Rap‐1b, a member of the RAS oncogene family [102]. These findings suggest that differential EV cargo distribution could serve as a minimally invasive biomarker for skin cancer screening, staging, and treatment response.

Similarly, EVs from patients with inflammatory skin diseases such as psoriasis contain altered cytokines, growth factors, and miRNAs, e.g., miR‐381‐3p [84]. While these candidates may reflect disease activity and tissue remodelling, independent validation is required before they can be considered robust biomarkers of disease severity or treatment response. Also, microbial EVs found in serum could serve as biomarkers for AD, given the substantial differences in microbial diversity between AD patients and healthy controls [85].

Overall, skin‐derived EVs as biomarkers promise to offer highly sensitive and specific information about diseases, potentially revealing markers earlier than conventional diagnostic techniques. This could lead to earlier interventions and better clinical outcomes. However, challenges in standardizing EV separation and characterization methods, and further research is necessary to confirm the functional relevance of specific EV cargo and to improve reproducibility in clinical settings. Advances in technologies such as RNA sequencing, proteomics, and nanoparticle tracking are expected to enhance the detection and profiling of skin‐derived EVs.

## 9 Therapeutic Application of EVs in Dermatology

EVs have garnered significant attention for their therapeutic potential, as they can cross biological barriers, mediate cell‐to‐cell communication, potentially deliver therapeutic cargo, and modulate immune responses. Stem cell‐derived EVs have shown great promise for treating various disorders and promoting tissue regeneration. These vesicles carry bioactive molecules that can influence biological processes, making them a potentially versatile therapeutic approach for numerous diseases.

EV biogenesis in skin involves multiple sphingomyelinases with distinct subcellular localizations and pH optima. nSMase has been linked to increased sEV release from senescent fibroblasts and to ceramide generation at neutral pH, whereas ASM contributes to ceramide‐rich microdomains at the plasma membrane and within endolysosomal compartments. In addition to its effects on sEV composition, ASM activity has been implicated in the plasma‐membrane budding and release of lEVs. Pharmacological ASM inhibitors, including functional inhibitors such as imipramine and amitriptyline, may therefore provide an experimentally tractable approach for modulating lEV release, although their effects are not necessarily specific to EV biogenesis [103]. Recent work inhibiting nSMase or ASM in human cells shows that these enzymes differentially affect EV protein and RNA cargo sorting, rather than simply increasing or decreasing EV numbers, underscoring that pharmacological targeting of sphingomyelinases may modulate EV quality as well as quantity [104].

EVs from human ADSCs are shown to mitigate senescence phenotype in human dermal fibroblasts, reducing markers such as senescence‐associated beta‐galactosidase (SA‐β‐Gal), ROS production, and senescence‐associated pathways involving p53, p16, and p21 [105]. Such findings support the potential of EV‐based anti‐ageing therapies, particularly in reversing or delaying cellular alterations that lead to skin degeneration, reduced collagen production, and impaired healing responses. Although preclinical data on stem cell‐derived EVs in skin ageing, inflammatory dermatoses, and chronic wounds are encouraging, current clinical evidence is restricted to small, often uncontrolled studies. This preclinical dominance, together with variable EV sourcing and characterization, means that many proposed therapeutic applications should still be regarded as experimental, and robust randomized controlled trials will be required to overcome publication bias and establish true efficacy and safety.

EVs also show therapeutic potential in inflammatory skin conditions like AD. Exosomes from human dermal neonatal fibroblasts promote skin‐barrier repair and suppress inflammation in atopic dermatitis through PPARα activation, highlighting their potential as a cell‐free therapeutic strategy [106]. Notably, subcutaneous injections of ADSC‐derived EVs in a mouse dermatitis model resulted in enhanced epidermal barrier function, reduced trans‐epidermal water loss, and increased skin hydration. These EVs also induced increased ceramide production and decreased levels of inflammatory cytokines, that is, IL‐4, IL‐5, IL‐13, TNF‐α, IFN‐γ, and IL‐17 [107]. Similarly, parenteral administration of human ADSC‐derived EVs also ameliorated AD symptoms and inflammatory markers in a murine model of the disease [108]. The ability of stem cell‐derived EVs to modulate immune responses and skin integrity positions them as a promising therapy for AD and potentially other inflammatory skin conditions.

EV‐based therapies are being actively explored to accelerate healing in chronic wounds, such as diabetic ulcers and burns. EVs carry growth factors, cytokines, and miRNAs that may contribute to signalling at the wound site and thereby support tissue repair and regeneration. For instance, hyaluronic acid‐containing EVs from human umbilical cord blood mononuclear cells have shown pro‐healing effects by promoting neovascularization and re‐epithelialization and by regulating key wound‐healing miRNAs [109]. Additionally, EVs carrying miR‐125b from young fibroblasts stimulate aged fibroblast migration and myofibroblast differentiation [110], while EVs from ADSCs loaded with miR‐10b enhance keratinocyte proliferation and migration, which are key steps in wound healing [111]. Furthermore, human umbilical cord blood mononuclear cells‐derived EVs carrying miR‐425‐5p improve endothelial cell survival and neovascularization via the PTEN/PI3K/AKT pathway [112].

EV‐mediated modulation may also contribute to tissue regeneration. MSC‐derived EVs have been reported to promote M2 macrophage polarization in diabetic wound models [113, 114, 115, 116]. However, MSC‐derived EVs have been shown to exhibit source‐dependent procoagulant activity associated with surface expression of phosphatidylserine and tissue factor, underscoring the need to evaluate the thrombogenic potential of EV‐based therapeutics during their development and clinical translation [117].

In summary, a growing body of evidence supports the therapeutic potential of EVs, particularly those derived from stem cells, across a range of applications, from anti‐ageing therapies to the treatment of inflammatory skin diseases and chronic wounds. Their ability to carry a bioactive cargo, modulate immune responses, and promote tissue regeneration positions EVs as a promising tool in regenerative medicine. However, additional research is required to improve the separation, characterization, and delivery of EVs, as well as to confirm their safety and effectiveness in clinical settings. Additionally, the current evidence base is dominated by in vitro and animal studies, with only early clinical exploration in selected areas such as skin rejuvenation and chronic wounds. Consequently, all EV‐based interventions discussed here should be regarded as experimental—preclinical or early clinical—rather than clinically established, and none currently meets the evidentiary threshold to be considered standard dermatological care.

It is worth noting that, in parallel with these preclinical and emerging clinical efforts, a growing number of cosmetic products are marketed as containing ‘exosomes’ or ‘EVs’ without clear information on source, separation or safety testing. This underscores the importance of adhering to MISEV‐compliant definitions and transparent reporting to distinguish rigorously characterized EV‐based interventions from poorly defined commercial formulations.

To clarify the current translational status of EV‐based therapies in dermatology, Table 1 summarizes the predominant evidence levels, translational readiness, and principal limitations across the major therapeutic areas discussed.

**TABLE 1:** Evidence level, translational status and principal limitations of extracellular vesicle–based therapeutic approaches in dermatology.

Therapeutic area	EV source (examples)	Main model types	Evidence level	Translational status	Main limitations
Skin ageing/senescence	ADSC‐derived EVs, MSC EVs	Human dermal fibroblasts in vitro; murine photoageing models; small human case series	In vitro, animal, early clinical	Experimental; early clinical exploration; not standard of care	Predominantly preclinical evidence; clinical studies are small and uncontrolled, with heterogeneous EV formulations.
Inflammatory skin diseases	Exosomes from human dermal neonatal fibroblasts; ADSC EVs	Human dermal fibroblasts, murine AD‐like models	In vitro, animal	Experimental; preclinical only	Evidence predominantly from preclinical models; heterogeneous EV sources and administration approaches; lack of controlled clinical studies; human efficacy and safety remain to be established.
Chronic wounds (diabetic, burn)	Umbilical‐cord blood mononuclear‐cell EVs; ADSC EVs; fibroblast EVs	Rodent wound models; small pilot clinical studies	Animal, early clinical	Experimental; limited clinical data	Heterogeneous wound models and EV preparations; clinical evidence is limited and outcomes are not standardized.
Skin cancer	Tumour‐derived EVs; stem cell EVs	In vitro melanoma/cSCC models; murine xenografts	In vitro, animal	Experimental; no clinical therapeutic applications yet	Predominantly mechanistic in vitro and animal evidence; clinical therapeutic efficacy has not been demonstrated.

## 10 Emerging Technologies and Approaches for EV Research in Dermatology

The impermeability of biological barriers, such as the skin, protects the body but also poses challenges for the effective delivery of therapeutic agents. EVs, with their natural biocompatibility and potential to serve as carriers of therapeutic cargo, offer a promising solution by enabling active permeability, targeted delivery, and protection of payloads from degradation [118]. Currently, drugs can be loaded into EVs using either the exogenous method, in which drugs are introduced directly into EVs, or the endogenous method, in which they are introduced into donor cells before EV release [119].

To improve the skin‐penetrating capacity of EVs, several innovative techniques have been developed. One such approach involves the use of microneedles (MNs) [120]. MNs facilitate the delivery of drugs and drug carriers, including EVs, directly to the dermis, improving drug absorption and therapeutic efficacy [121]. Hydrogel‐forming MNs are particularly promising due to their high drug‐loading capacity and controlled release. Their non‐toxic, painless nature makes EVs highly attractive for long‐term drug delivery applications [122, 123]. Studies have also demonstrated that MNs can enhance the biological functions of stem cell‐derived EVs, such as promoting collagen and elastin synthesis and fibroblast proliferation, making them an effective method for improving skin regeneration [124].

MN patches containing EVs have been developed to deliver therapeutic agents for wound healing, particularly diabetic wounds, in a controlled manner. These patches maintain EV biological activity to promote cell migration and angiogenesis, thereby significantly aiding wound repair. For instance, a methacrylated gelatin (GelMA)/PEGDA hydrogel MN patch loaded with human umbilical vein endothelial cells‐derived EVs has been shown to promote full‐thickness wound healing in diabetic mice by stimulating cellular processes in the deeper layers of the skin [125].

Finally, 3D bioprinting is poised to revolutionize skin research and EV‐based therapies. The use of 3D bioprinting in conjunction with EV‐loaded hydrogels has shown great promise in creating scaffolds for wound healing. These hydrogels, which provide both mechanical support and bioactive properties, can be printed with EVs to enhance tissue regeneration in wound treatment [126]. One study demonstrated that dynamic culture in a perfusion bioreactor could significantly increase EV production from endothelial cells, suggesting a scalable approach for producing EVs in large quantities for therapeutic applications [127]. This technology holds immense potential for developing advanced treatments for chronic wounds and tissue regeneration.

In summary, integrating EVs with emerging technologies such as MNs and 3D bioprinting offers exciting opportunities to advance skin research and develop novel dermatological treatment modalities for more effective, non‐invasive EV‐based therapies. Further studies and development in this field are critical to unlock the full therapeutic potential of EVs in research and clinical dermatology.

## 11 Major Open Questions

Despite the remarkable progress in understanding the roles of EVs in skin biology, several fundamental questions remain unanswered. Addressing these will be essential for translating EV research into meaningful clinical applications in dermatology. A concise summary of key recommendations to guide future efforts in this direction is provided in Box 2.

First, how can EV separation and characterization be standardized to ensure reproducibility? The field currently lacks universally adopted protocols, and discrepancies in methods for EV purification, quantification, and molecular profiling hinder cross‐study comparisons. Adopting and enforcing the MISEV2023 guideline [7] across dermatological EV research, along with the development of reference materials and inter‐laboratory quality controls, is a necessary step toward reliable, reproducible findings.

Second, what determines the cargo specificity of skin‐derived EVs, and how is this process regulated? While it's clear that EV content varies with cell type and stimulus, the molecular mechanisms underlying selective cargo loading remain poorly understood. Using high‐throughput multi‐omics profiling of EVs from different skin cell types and conditions—along with functional validation in realistic models such as 3D skin equivalents—will be crucial for uncovering how EV cargo is selected and how it influences intercellular communication in the skin.

Third, can EV‐based liquid biopsies become viable diagnostic tools in dermatology? Biofluid‐derived EVs, including those obtained from sweat and blood, are being investigated as minimally invasive biomarkers, but their clinical utility remains unestablished.

Fourth, what is the long‐term safety and efficacy of EV‐based therapies? Although stem cell‐derived EVs have shown encouraging results in preclinical models of wound healing and skin ageing, evidence from rigorous clinical trials remains scarce. Studies evaluating the pharmacokinetics, biodistribution, immunogenicity, and potential off‐target effects of therapeutic EVs in human skin are urgently needed before clinical translation.

A further conceptual challenge is quantifying how often EV luminal cargo reaches the cytosol of skin cells versus effects mediated by EV surface or endosomal signalling, as recent quantitative work suggests that true cytosolic delivery may be infrequent.

Finally, how can emerging delivery technologies be optimized for EV‐based treatments? Microneedle arrays and 3D‐bioprinted scaffolds offer exciting avenues for controlled EV delivery to the skin, yet many technical challenges persist regarding scalability, EV stability during formulation, and controlled release kinetics. Systematic optimization through interdisciplinary collaborations between bioengineers, material scientists, and dermatologists will be instrumental in advancing these platforms toward clinical use.

BOX 2 **Opinions, considerations and future perspectives.**

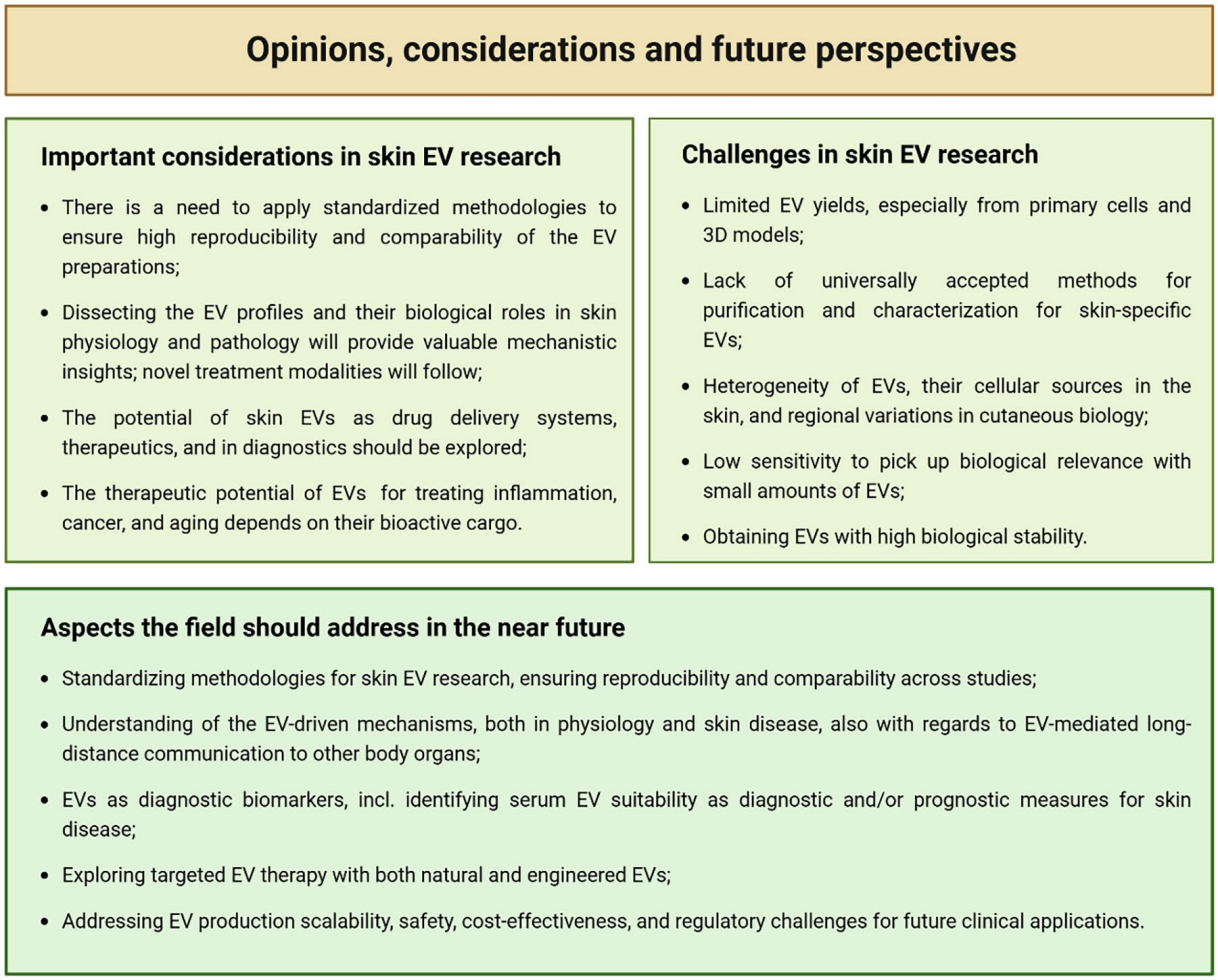

## 12 Conclusions and Perspectives

This review has highlighted the multifaceted roles of EVs in skin biology, from maintaining homeostasis and modulating immune responses to driving pathological processes such as ageing, cancer progression, and chronic inflammation. Several key take‐home messages emerge from this synthesis. First, skin should not be viewed as an isolated organ but rather as an integral component of a highly interconnected physiological network. Through its extensive vascular, lymphatic, and neuronal connections, the skin continuously exchanges signals with other organs, enabling EV‐mediated intercellular communication at both local and systemic levels. Consequently, skin‐derived EVs not only reflect local tissue physiology and pathology but may also provide insights into systemic health, while circulating EVs originating from distant organs can influence skin homeostasis and disease.

A second major concept is that EVs serve as versatile mediators of intercellular communication in the skin, carrying cell‐type‐specific and stimulus‐dependent cargo that influences gene expression, immune regulation, and tissue remodelling across all skin compartments. In skin ageing and cellular senescence, EVs function both as propagators of age‐related signalling—through SASP components and senescence‐associated miRNAs—and as potential quality control mechanisms for mitochondrial disposal, opening avenues for anti‐ageing interventions. In the tumour microenvironment, EVs from melanoma and other skin cancers actively shape immune evasion, pre‐metastatic niche formation, and stromal reprogramming, underscoring their value as targets for early diagnosis and therapeutic intervention. Furthermore, EVs play dual roles in inflammatory skin diseases such as atopic dermatitis and psoriasis, both amplifying pathogenic immune loops and offering potential as immunomodulatory therapeutic agents. Emerging technologies—including EV‐loaded microneedle patches, 3D‐bioprinted scaffolds, and engineered EV formulations—hold significant promise for non‐invasive, targeted delivery of EV‐based therapies to the skin.

In the near term, progress will depend on standardized EV separation and reporting, quantitative functional assays, independent replication, and well‐powered human observational cohorts. Medium‐term priorities include validated biomarkers, scalable manufacturing, and rigorous safety studies. Controlled clinical trials and advanced delivery platforms should be regarded as longer term objectives rather than established applications. This staged agenda reflects the current maturity of the field and defines the evidence required to determine whether EV‐based diagnostics or treatments can progress toward routine dermatological care. Interdisciplinary efforts, such as those supported by the COST Action NETSKINMODELS, will be crucial to unify research practices and close the gap between laboratory discoveries and clinical applications.

## Author Contributions

M.C. conceived the article, coordinated the work, acted as the leading contact and served as the main corresponding author. M.C. and N.A. jointly supervised the development of the manuscript. All authors contributed substantially and equally to the writing, critical revision and intellectual content of the review. M.C., M.N.‐A. and D.G.‐O. had leading roles in preparing figures and boxes. All authors reviewed and approved the final version of the manuscript.

## Funding

D.G.‐O. acknowledges funding from the FENG.02.02‐IP.05‐0332/23 project, carried out within the First TEAM programme of the Foundation for Polish Science, co‐financed by the European Union under the European Funds for Smart Economy 2021‐2027 (FENG). A.O. and D.Á. acknowledge funding from the National Research, Development and Innovation Office (Grant IDs: 134235, 152241, EKÖP‐25‐4‐I‐DE‐225) and from the University of Debrecen (‘Bridging Fund’ of the Faculty of Medicine, ‘University of Debrecen Scientific Research Bridging Fund’ [‘DETKA’] and ‘University of Debrecen Program for Scientific Publication’). PP acknowledges funding from the MCIN/AEI/10.13039/501100011033 (Grant No. PID2023‐147966OB‐I00) and the Generalitat Valenciana (Grant No. Prometeo CIPROM22/042). NA acknowledges the University of Oulu Profi8 Health Dimensions program and Research Council of Finland Decision 365202, and Business Finland (Funding Decision 4417/31/2025) for their support. This work was supported by the Italian Ministry of Health with Current Research funds to the following authors: AP, SPB and AM.

## Ethics Statement

The authors have nothing to report.

## Consent

The authors have nothing to report.

## Conflicts of Interest

A.O. provides consultancy services to QIMA Monasterium (previously known as Monasterium Laboratory Skin & Hair Research Solutions GmbH), Münster, Germany. The author S.O. is also co‐owner of Regensis Health Co., a consultancy company specializing in cell‐based regenerative therapies, including exosomes. This paper is academic in nature and independent, with no commercial or financial interests tied to the company. No conflicts of interest are declared beyond this professional affiliation.

## Data Availability

Data sharing not applicable to this article as no datasets were generated or analysed during this study.
